# Supplementary material for: Nutrient Profiles of Commercially Produced Complementary Foods Available in Burkina Faso, Cameroon, Ghana, Nigeria and Senegal
Source: Nutrients. 2023 May 11;15(10):2279. doi: 10.3390/nu15102279 (PMC10222290; doi:10.3390/nu15102279)
Supplement: Supplementary file 1 [file nutrients-15-02279-s001.zip › Supplementary Table S1.pdf]

**Supplementary Table S1**: WHO/FAO (2004) recommended nutrient intakes (RNI) for iron, calcium and zinc.	
Calcium RNI for 6-12 months of age [1]	400 mg/day
Calcium RNI for 12-36 months of age [1]	500 mg/day
Iron RNI for 6-12 months of age	9.3 mg/day
Iron RNI for 12-36 months of age	5.8 mg/day
Zinc RNI for 12-36 months of age [2]	4.1 mg/day

[1] 10% bioavailability
[2] Moderate bioavailability
