# Supplementary material for: Nutrient Profiles of Commercially Produced Complementary Foods Available in Burkina Faso, Cameroon, Ghana, Nigeria and Senegal
Source: Nutrients. 2023 May 11;15(10):2279. doi: 10.3390/nu15102279 (PMC10222290; doi:10.3390/nu15102279)
Supplement: Supplementary file 1 [file nutrients-15-02279-s001.zip › Supplementary Table S3.pdf]

Supplementary Table S3. Total sugar, sodium, protein and total fat content per 100g of commercially produced complementary food products, by country[1.]

Product category	n[2]	Total sugar per 100g (g)	n[2]	Sodium per 100g (mg)	n[2]	Protein per 100g (g)	n[2]	Total fat per 100g (g)
**Burkina Faso (n=20)**								
1.1 Dry or instant cereals/starches	7	32.0 [27.6-36.0]	20	135 [110-176]	17	15.0 [14.6-15.0]	20	9.7 [6.2-10.0]
**Cameroon (n=52)**								
1.1 Dry or instant cereals/starches	23	32.2 [23.0-35.0]	39	130 [100-160]	44	15.0 [14.0-15.7]	42	4.3 [3.5-9.5]
2.1 Dairy-based desserts and cereal products	1	7.2 [--]	1	30 [--]	1	2.7 [--]	1	2.5 [--]
2.2 Fruit puree	2	9.4 [8.7-10.0]	2	7 [3-10]	2	0.5 [0.4-0.5]	2	0.1 [0.0-0.1]
2.4 Vegetable puree with cereals	1	4.8 [--]	1	60 [--]	1	2.3 [--]	1	2.3 [--]
2.6 Pureed meal with meat/fish in product name	0	-	0	-	1	3.2 [--]	1	0.9 [--]
2.7 Pureed meal with meat/fish not in product name	0	-	0	-	1	2.8 [--]	1	0.9 [--]
**Ghana (n=127)**								
1.1 Dry or instant cereals/starches	24	26.7 [12.9-32.9]	46	135 [12-191]	49	10.3 [6.7-15.0]	48	5.3 [3.3-9.0]
2.1 Dairy-based desserts and cereal products	12	7.3 [1.1-15.6]	13	40 [24-51]	14	2.5 [1.7-3.0]	14	2.0 [1.3-3.0]
2.2 Fruit puree	31	8.9 [7.2-10.8]	42	8 [1-12]	42	0.6 [0.4-1.0]	42	0.1 [0.0-0.7]
2.3 Vegetable only puree	2	4.1 [3.7-4.4]	4	16 [5-33]	4	3.1 [0.9-5.5]	4	0.1 [0.0-0.1]
2.4 Vegetable puree with cereals	0	-	2	4 [1-7]	2	0.9 [0.4-1.3]	2	0.2 [0.1-0.3]
2.5 Pureed meal with cheese	1	3.0 [--]	1	127 [--]	1	0.5 [--]	1	0.0 [--]
2.6 Pureed meal with meat/fish in product name	1	0.7 [--]	1	40 [--]	1	3.5 [--]	1	1.7 [--]
2.7 Pureed meal with meat/fish not in product name	5	2.3 [1.7-2.8]	5	12 [12-30]	5	2.9 [2.7-3.2]	5	2.3 [1.7-2.4]
4.3 Snacks and finger foods	4	21.1 [17.7-27.7]	4	120 [60-143]	4	6.7 [3.4-7.9]	4	10.4 [5.4-11.5]
**Nigeria (n=109)**								
1.1 Dry or instant cereals/starches	30	32.1 [24.3-34.1]	59	132 [76-191]	67	14.3 [9.0-15.5]	70	8.2 [3.2-10.0]
2.1 Dairy-based desserts and cereal products	3	6.7 [6.6-7.5]	3	1 [0.0-32]	3	2.4 [1.2-2.9]	3	2.0 [0.1-2.5]
2.2 Fruit puree	10	10.2 [9.9-10.9]	11	0 [0-1]	11	0.6 [0.3-0.9]	11	0.2 [0.0-0.4]
2.4 Vegetable puree with cereals	1	4.7 [--]	1	3 [--]	1	2.4 [--]	1	0.9 [--]
2.6 Pureed meal with meat/fish in	1	4.7	1	15	1	1.8	1	0.9

product name		[--]		[--]		[--]		[--]
2.7 Pureed meal with meat/fish not in product name	1	0.2 [--]	1	48 [--]	1	2.6 [--]	1	0.6 [--]
4.3 Snacks and finger foods	10	28.0 [14.3-43.1]	12	181 [6-204]	12	6.0 [0.0-7.5]	12	10.2 [0.0-22.0]
**Senegal (n=333)**								
1.1 Dry or instant cereals/starches	43	25.1 [17.3-32.7]	73	76 [17-130]	75	11.0 [9.0-14.0]	76	3.2 [1.4-7.8]
2.1 Dairy-based desserts and cereal products	30	8.4 [7.6-9.2]	30	40 [30-40]	30	2.9 [2.6-3.2]	30	3.1 [2.7-3.2]
2.2 Fruit puree	77	10.0 [9.0-11.0]	79	3 [3-5]	80	0.5 [0.4-0.5]	80	0.2 [0.1-0.5]
2.3 Vegetable only puree	29	2.1 [0.9-3.1]	30	19 [10-28]	30	1.1 [0.8-1.6]	30	0.5 [0.2-1.1]
2.4 Vegetable puree with cereals	21	2.2 [1.8-3.4]	21	20 [13-31]	21	2.0 [1.3-2.4]	21	1.7 [1.1-1.9]
2.5 Pureed meal with cheese	3	1.5 [1.0-1.6]	3	80 [30-80]	3	3.3 [3.2-4.5]	3	2.7 [2.4-2.8]
2.6 Pureed meal with meat/fish in product name	1	0.5 [--]	1	32 [--]	3	3.4 [3.3-3.5]	3	1.3 [1.0-1.4]
2.7 Pureed meal with meat/fish not in product name	26	1.9 [1.6-2.3]	26	26 [20-30]	27	2.7 [2.3-2.9]	27	1.9 [1.6-2.0]
3.1 Chunky meal with meat/fish/cheese	24	1.4 [1.1-1.7]	24	99 [94-110]	24	2.8 [2.5-3.0]	24	2.2 [2.0-2.6]
3.2 Chunky meal with vegetables	25	1.8 [1.3-2.8]	25	97 [60-99]	25	2.3 [1.6-2.7]	25	2.0 [1.8-2.7]
4.3 Snacks and finger foods	10	17.3 [16.0-25.5]	10	50 [40-124]	10	6.4 [6.1-8.5]	10	13.6 [12.0-23.2]

[1]Values are presented as median [interquartile range]

[2]Products without relevant nutrient content declarations on label are excluded
