# Supplementary material for: Nutrient Profiles of Commercially Produced Complementary Foods Available in Burkina Faso, Cameroon, Ghana, Nigeria and Senegal
Source: Nutrients. 2023 May 11;15(10):2279. doi: 10.3390/nu15102279 (PMC10222290; doi:10.3390/nu15102279)
Supplement: Supplementary file 1 [file nutrients-15-02279-s001.zip › Supplementary Table S4.pdf]

Supplementary Table S4: Calcium, iron and zinc content per 100g of commercially products complementary foods, by country.

Product category	Serving size (g), median (IQR)	Calcium content declared (n)	Calcium (mg), median (IQR)	Iron content declared (n)	Iron (mg), median (IQR)	Zinc content declared (n)	Zinc (mg), median (IQR)
**Burkina Faso**							
1 Dry, powdered and instant cereal/starchy food (n=20)	47 (44 - 50)	20	450 (322 - 613)	20	9.5 (7.5 - 10.7)	16	4.5 (4.3 - 5.5)
**Cameroon**							
1 Dry, powdered and instant cereal/starchy food (n=46)	45 (35 - 49)	38	450 (388 - 662)	42	8.0 (5.7 - 10.5)	27	5.5 (4.5 - 5.5)
2 Soft-wet spoonable, ready-to-eat foods (purees) (n=6)	200 (148 - 200)	2	65 (63 - 67)	1	0.9 (--)	0	--
**Ghana**							
1 Dry, powdered and instant cereal/starchy food (n=54)	32 (20 - 50.0)	26	392 (316 - 450)	35	10.0 (7.6 - 15.0)	28	5.5 (4.5 - 6.1)
2 Soft-wet spoonable, ready-to-eat foods (purees) (n=69)	130 (125 - 205)	10	74 (26 - 435)	7	4.8 (2.1 - 6.0)	4	2.5 (1.8 - 3.2)
4 Dry finger foods and snacks (n=4)	8 (5 - 8)	2	304 (300 - 307)	2	5.8 (5.6 - 5.9)	1	4.0 (--)
5 Juices and other drinks (n=4)	237	0	--	0	--	0	--
**Nigeria**							
1 Dry, powdered and instant cereal/starchy food (n=75)[1]	30 (25 - 50)	55	450 (226 - 511)	62	10.5 (6.9 - 14.4)	42	5.0 (2.6 - 7.0)
2 Soft-wet spoonable, ready-to-eat foods (purees) (n=17)	100 (100 - 120)	4	42 (12 - 71)	3	0.4 (0.3 - 0.7)	0	--
4 Dry finger foods and snacks (n=14)	15 (7 - 50)	5	390 (286 - 390)	5	11.0 (7.0 - 20.0)	1	2.2
5 Juices and other drinks (n=2)	118.0	1	0.0 (--)	1	0.0 (--)	1	0.0 (--)
**Senegal**							
1 Dry, powdered and instant cereal/starchy food (n=76)	44 (27 - 50)	50	306 (190 - 437)	68	9.0 (7.6 - 11.3)	42	4.5 (2.2 - 7.0)
2 Soft-wet spoonable, ready-to-eat foods (purees) (n=194)	100 (100 - 120)	26	96 (65 - 100)	3	1.0	3	1.5 (1.1 - 1.5)
3 Meals (n=49)	250 (235 - 250)	3	60 (60 - 65)	1	0.9 (--)	0	--
4 Dry finger foods and snacks (n=10)	10 (9 - 12)	2	320	3	7.0 (6.8 - 7.3)	0	--
5 Juices and other drinks (n=19)	--[2]	16	72 (69 - 77)	16	1.10 (0.7 - 1.3)	16	0.3 (0.3 - 0.4)

[1] Three products excluded from the micronutrient analyses due to unclear iron/calcium/zinc content values reported on labels
[2] No products declared serving size
